# Inflammatory and Metabolic Alterations of Kager's Fat Pad in Chronic Achilles Tendinopathy

**DOI:** 10.1371/journal.pone.0127811

**Published:** 2015-05-21

**Authors:** Jessica Pingel, M. Christine H. Petersen, Ulrich Fredberg, Søren G. Kjær, Bjørn Quistorff, Henning Langberg, Jacob B. Hansen

**Affiliations:** 1 Department of Exercise and Nutrition, University of Copenhagen, Copenhagen, Denmark; 2 Department of Biology, University of Copenhagen, Copenhagen, Denmark; 3 Department of Biomedical Sciences, University of Copenhagen, Copenhagen, Denmark; 4 Diagnostic Centre, Silkeborg Regional Hospital, Silkeborg, Denmark; 5 CopenRehab, Department of Public Health, University of Copenhagen, Copenhagen, Denmark; Queen Mary University of London, UNITED KINGDOM

## Abstract

**Background:**

Achilles tendinopathy is a painful inflammatory condition characterized by swelling, stiffness and reduced function of the Achilles tendon. Kager’s fat pad is an adipose tissue located in the area anterior to the Achilles tendon. Observations reveal a close physical interplay between Kager’s fat pad and its surrounding structures during movement of the ankle, suggesting that Kager’s fat pad may stabilize and protect the mechanical function of the ankle joint.

**Aim:**

The aim of this study was to characterize whether Achilles tendinopathy was accompanied by changes in expression of inflammatory markers and metabolic enzymes in Kager’s fat pad.

**Methods:**

A biopsy was taken from Kager’s fat pad from 31 patients with chronic Achilles tendinopathy and from 13 healthy individuals. Gene expression was measured by reverse transcription-quantitative PCR. Focus was on genes related to inflammation and lipid metabolism.

**Results:**

Expression of the majority of analyzed inflammatory marker genes was increased in patients with Achilles tendinopathy compared to that in healthy controls. Expression patterns of the patient group were consistent with reduced lipolysis and increased fatty acid β-oxidation. In the fat pad, the pain-signaling neuropeptide substance P was found to be present in one third of the subjects in the Achilles tendinopathy group but in none of the healthy controls.

**Conclusion:**

Gene expression changes in Achilles tendinopathy patient samples were consistent with Kager’s fat pad being more inflamed than in the healthy control group. Additionally, the results indicate an altered lipid metabolism in Kager’s fat pad of Achilles tendinopathy patients.

## Introduction

Adipose tissue has multiple functions including insulation and structural support, but is first and foremost a key metabolic tissue with profound influence on whole-body homeostasis [1]. Two main types of adipose tissue exist: white adipose tissue, which is the largest energy store of the body, and brown adipose tissue that is specialized in energy dissipation through uncoupled respiration. Adipocytes secrete adipokines, cytokines, and growth factors, acting in endocrine, paracrine or autocrine manners, thereby influencing appetite, insulin sensitivity, metabolism of other tissues and processes like healing, inflammation and tissue degradation [1–4].

Kager’s fat pad, also described as the pre-Achilles fat pad, is a sharply enclosed radiolucent triangle delineated by the flexor halluces longus muscle and tendon (anterior border), the superior cortex of the calcaneus (inferior border) and the Achilles tendon (posterior border) [5]. It has been suggested that the flexor halluces longus part of Kager’s fat pad contributes to moving the bursal wedge during plantar flexion, that the Achilles-associated part protects blood vessels passing through it to supply the Achilles tendon, and that the region near the bursal wedge minimizes pressure changes in the retrocalcaneal bursa [6]. Changes in Kager’s fat pad have been observed in a variety of conditions, including cortical destruction of the calcaneus mediated by either a tumor or infection, Os trigonum syndrome [5], accessory soleus muscle [7] and fibroma of the tendon sheath [8]. Radiographical changes in Kager’s fat pad can signal the presence of abnormality [5]. Abnormal conditions involving the posterior ankle may result in increased and ill-defined soft tissue density in Kager’s fat pad or the obliteration of its borders. Additionally, patients with chronic pain in the Achilles tendon, Achilles tendinopathy (AT), often show an increased soft tissue density simultaneously with an increased thickness of the Achilles tendon. Despite the common occurrence of pain symptoms and soft tissue alterations in Kager’s fat pad, this region has not been investigated, and detailed knowledge of its role in the pathophysiology of the Achilles tendon is sparse.

Another fat pad that has received more attention is the infrapatellar fat pad (IFP), also known as Hoffa’s fat pad. Chronic edema of the IFP is the key pathology of Hoffa’s disease, the edema leading to soft tissue impingement, ischemia, and in the long-term lipomatous tissue necrosis [9,10]. IFP contains inflammatory cells and is a source of adipokines, cytokines and growth factors, which might impact disease [11]. Several inflammatory mediators secreted from adipose tissue, including interleukin-1α (IL-1α), IL-1β, IL-6, IL-10, tumor necrosis factor-α (TNF-α), monocyte chemotactic protein-1 (MCP1) and prostaglandin E2, have been shown to be involved in joint diseases [12–14]. Furthermore, one study showed that adiponectin (ADIPOQ) induces matrix metalloproteinase-1 and IL-6 expression in synovial fibroblasts [15]. Thus, ADIPOQ might have pro-inflammatory functions in joint diseases [16]. These findings underline that, besides being a reservoir for storage of excess calories, adipose tissue plays an important role in the process of inflammation [12]. Whether these inflammatory cells and mediators are also present in Kager’s fat pad and whether the potential inflammatory state of the fat pad may influence pain and other symptoms of AT patients is still unclear.

The aim of the present study was to analyze gene expression changes in Kager’s fat pad of chronic AT patients. Our focus was on genes encoding macrophage markers, adipokines, cytokines and growth factors as well as genes involved in lipid and energy metabolism. We provide evidence that patient-derived fat pads display a remarkable inflammatory profile and altered expression of a number of metabolism-related genes.

## Materials and Methods

### Subjects

The subjects were fully informed and gave their written consent to participate in adherence to the declaration of Helsinki. The study was approved by the Ethical Committee of the capital region of Copenhagen, Denmark (H-1-2009-114). Thirty-one AT patients were recruited for this study. One AT patient had to be excluded due to failure in the process from biopsy to cDNA. In addition, 13 healthy control subjects were recruited. The AT patients were all recreational athletes or persons with physical work with a long history of chronic Achilles tendon pain (subject characteristics are shown in Table 1), on whom conventional conservative treatments, like eccentric training programs, non-steroidal anti-inflammatory drug medication, and corticosteroid injections, had been tried with no lasting effect. A window of at least 6 months between the most recent last intake of these medications and the present study was ensured in all cases. Control subjects had no previous history of symptoms of Achilles tendon or Kager’s fat pad pain sensation and were without acute or chronic diseases. All AT patients were recruited from the Diagnostic Centre, Silkeborg Regional Hospital, Denmark.

**Table 1:** Subject characteristics.

	Subjects	Sex	Age	Height	Weight	BMI	Duration of symptoms
	(n)	(F/M)	(years)	(m)	(kg)		(mo.)
Controls	13	7/6	29	1.73	71.1	23.5	_
			(19–48)	(1.6–1.88)	(51–94)	(19.9–29.0)	
Patients	30	10/20	49	1.78	91.2	28.8	40
			(31–67)	(1.6–1.96)	(66.7–122)	(22.5–38.5)	(9–156)

Values are presented as mean (min—max).

F, female; M, male; BMI, body mass index; mo., months.

### Design and biopsy procedure

To ensure that all participants were in a resting state, they were asked to abstain from any kind of exercise 24 h prior to the experiment. After arrival to the clinic, body weight and height were recorded before preparation for biopsy. A biopsy of Kager’s fat pad was taken from the subjects placed in a supine position. The subjects were locally anesthetized under ultrasound guidance with 2 ml 1% lidocaine in the peritendinous space from both the medial and lateral side of the tendon. An ultrasound guided biopsy of Kager’s fat pad was taken from the middle of the Kager’s triangle with a semi-automatic biopsy needle [14 GA 9 cm (Angiotech)]. The samples were snap-frozen in liquid nitrogen and stored at -80°C for later RNA isolation.

### Tissue lysis and homogenization

Adipose tissue biopsies were kept on dry ice during most of the lysis procedure. The tissue was rinsed shortly in phosphate-buffered saline (Life Technologies) to remove excess blood, placed in 2-ml capped micro tubes (Sarstedt) with ceramic beads (Precellys) on dry ice. TRI Reagent (Sigma-Aldrich) (1 ml) was quickly added and the tissue was lysed and homogenized using a Precellys 24 instrument (Bertin Technologies). All lysates were quickly transferred to Eppendorf tubes on dry ice and stored at -80°C until RNA was isolated.

### RNA purification and reverse transcription

Total RNA was isolated according to the instructions of the manufacturer (Sigma-Aldrich). Briefly, after homogenization in TRI Reagent, total RNA was isolated by phase-separation after addition of 0.2 ml chloroform. RNA was precipitated from the aqueous phase by addition of 0.5 ml isopropanol. The resulting RNA pellet was washed in 1 ml 75% ethanol and dissolved in 10 μl diethyl pyrocarbonate-treated H_2_O and the RNA samples were frozen at -80°C. Reverse transcription was carried out essentially as described [17]. Briefly, total RNA was treated with RQ1 DNase (Promega) for 30 min at 37°C, followed by heat inactivation for 5 min at 75°C. Reactions were performed in 25 μl containing 1x 1st Strand Buffer (Life Technologies), 0.1 μg random hexamers (Bioline), 0.9 mM of each dNTP (Sigma-Aldrich), 20 units of RNaseOUT (Sigma-Aldrich), 1 μg of total DNase-treated RNA and 200 units of Moloney murine leukaemia virus reverse transcriptase (Life Technologies). Reactions were left for 10 min at room temperature, followed by incubation at 37°C for 1 h. After cDNA synthesis, reactions were diluted with 50 μl of nuclease-free water and frozen at -80°C.

### Reverse transcription-quantitative polymerase chain reaction (RT-qPCR)

Diluted cDNA (1.5 μl) in a final PCR mixture volume of 20 μl, was analyzed by RT-qPCR using the Stratagene Mx3000P QPCR System (Agilent Technologies). Besides the cDNA, each PCR mixture contained 10 μl of 2x SensiFAST SYBR Lo-Rox Kit (Bioline), 2 pmol of each primer (forward and reverse) (0.1 μM, final concentration) and UltraPURE H_2_O. PCR was carried out in 96-well plates, where each sample was analyzed in duplicate using the following cycling conditions: 95°C for 10 min, then 40 cycles of 95°C for 15 s, 55°C for 30 s, and 72°C for 15 s, followed by 95°C for 1 min, 60°C for 30 s, and 95°C for 30 s. Target gene mRNA expression was normalized to the stably expressed TATA-binding protein (*TBP*) mRNA using the formula 0.5^(target C_t_—normalizer C_t_). The normalized values are designated the relative mRNA expression in the present study. Primers used for RT-qPCR are listed in S1 Table. Primers were designed using the Primer-BLAST tool from NCBI and were, when possible, designed to span an exon-exon junction. Primer efficacy was validated by serial dilution of template and melting curve analysis was used to confirm the presence of a single amplicon.

### Statistics

**Fig 1:**
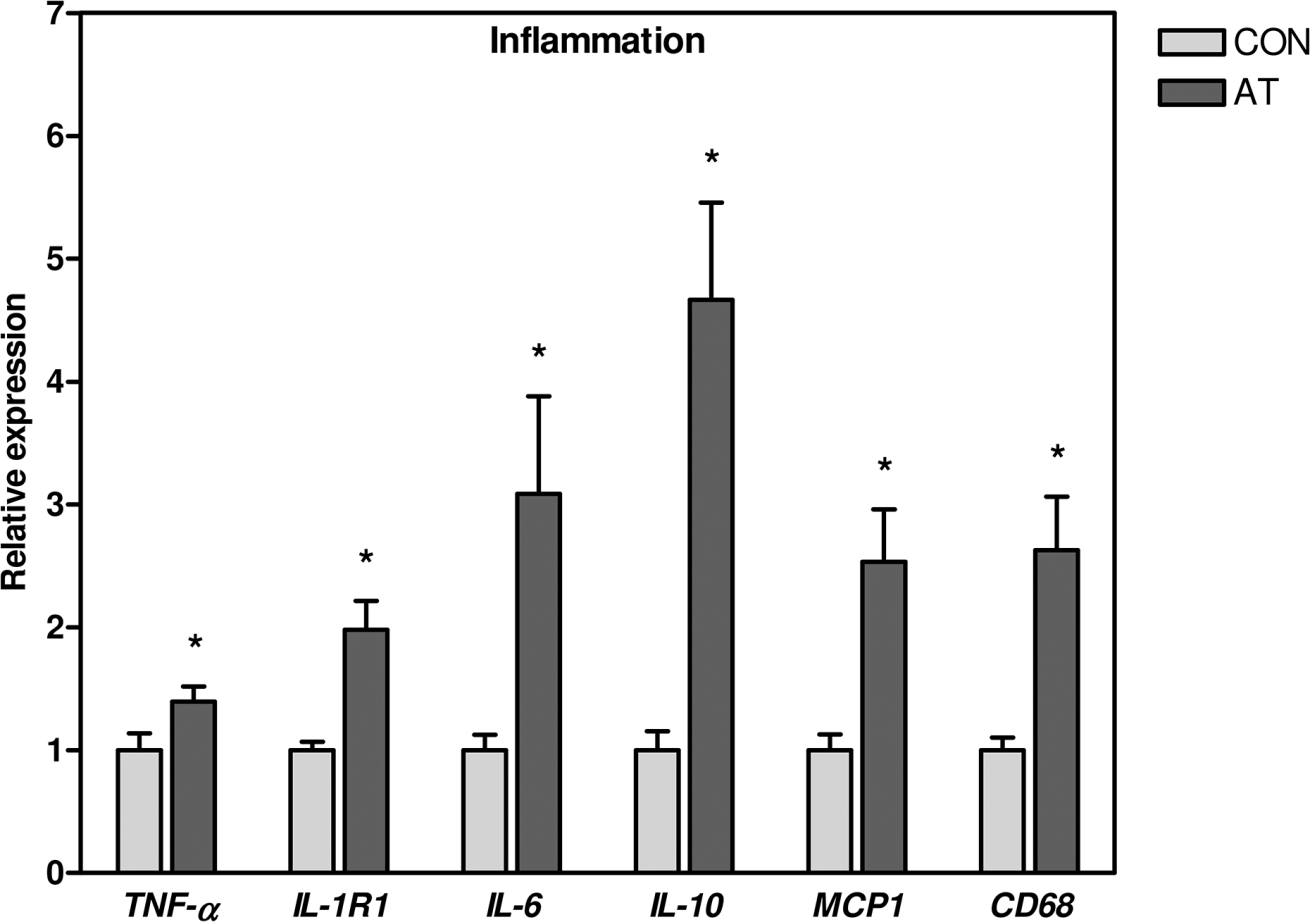
Inflammatory markers. mRNA levels of the inflammatory markers *TNF-α*, *IL-1R1*, *IL-6*, *IL-10* and *MCP1* and of the macrophage marker *CD68* were significantly elevated in the Kager’s fat pad of AT patients (n = 30) compared to healthy CON subjects (n = 13). The expression of target gene mRNA was normalized to the expression of *TBP* mRNA. Normalized mRNA expression for the control subjects was set to 1. * *p* < 0.05 (compared to CON). *TNF-α*, tumor necrosis factor-α; *IL-1R1*, interleukin-1 receptor 1; *IL-6*, interleukin-6; *IL-10*, interleukin-10; *MCP1*, monocyte chemotactant protein-1; *CD68*, cluster of differentiation 68; CON, control; AT, Achilles tendinopathy; *TBP*, TATA-binding protein.

**Fig 2:**
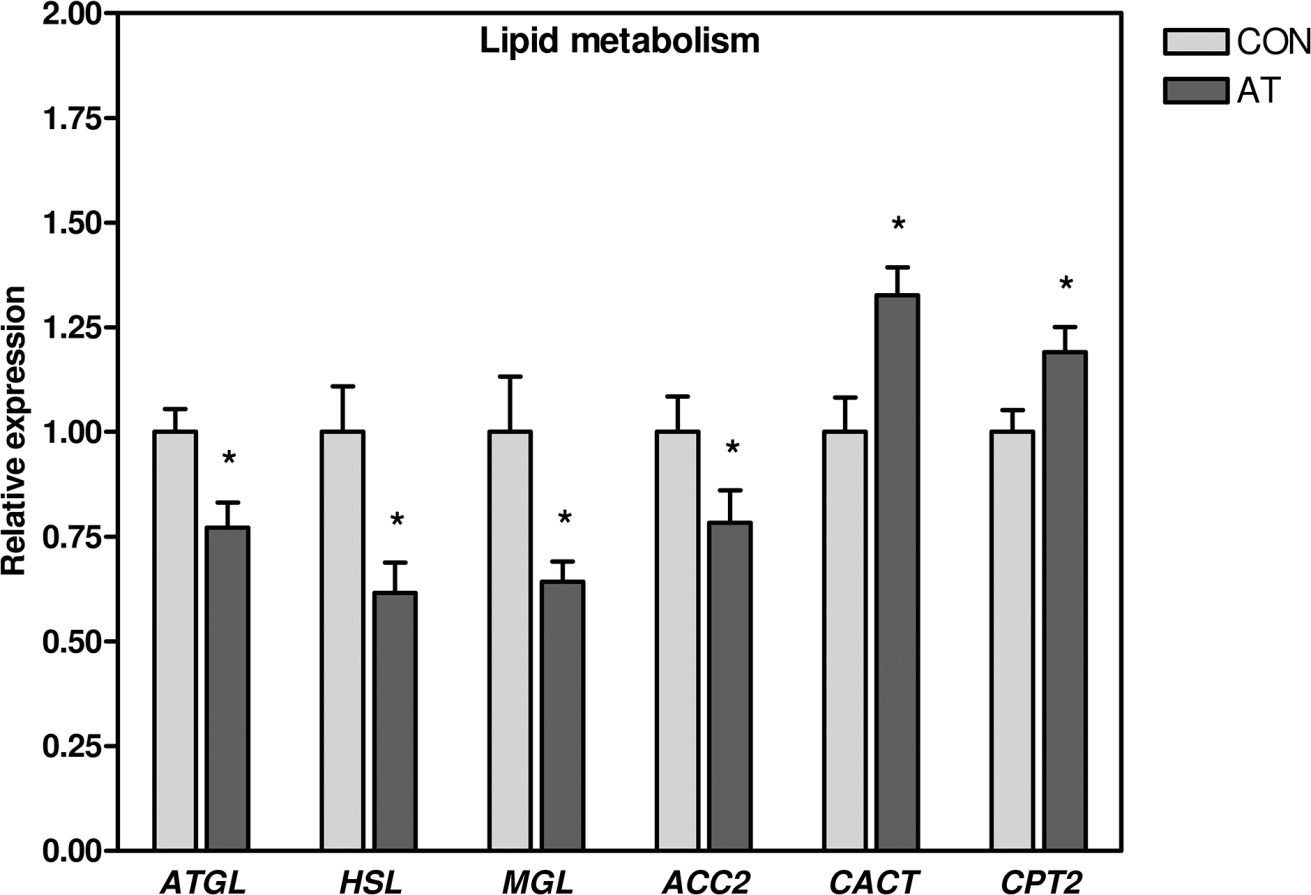
Lipid metabolism. mRNA levels of *ATGL*, *HSL*, *MGL*, and *ACC2* were significantly lower in Kager’s fat pad of AT patients (n = 30) compared to healthy CON subjects (n = 13, except *ATGL* and *HSL*: n = 12), while the mRNA expression of *CACT* and *CPT2* was significantly elevated. The expression of target gene mRNA was normalized to the expression of *TBP* mRNA. Normalized mRNA expression for the control subjects was set to 1. * *p* < 0.05 (compared to CON). *ATGL*, adipose triglyceride lipase; *HSL*, hormone-sensitive lipase; *MGL*, monoacylglycerol lipase; *ACC2*, acetyl-CoA carboxylase 2; AT, Achilles tendinopathy; CON, control; *CACT*, carnitine-acylcarnitine translocase; *CPT2*, carnitine palmitoyltransferase 2; *TBP*, TATA-binding protein.

**Fig 3:**
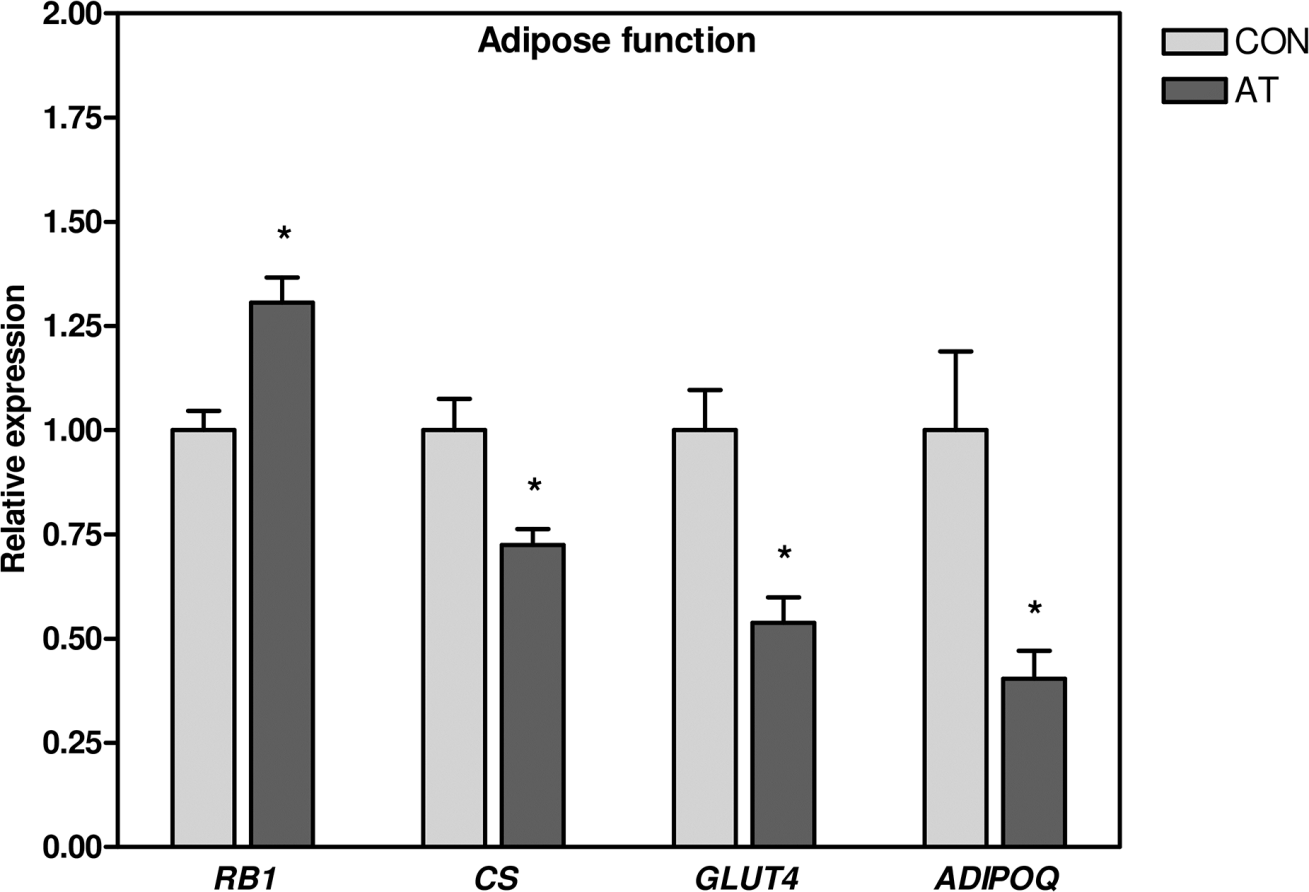
Energy metabolism, *GLUT4* and *ADIPOQ*. mRNA levels of *CS*, *GLUT4* and *ADIPOQ* were significantly lower, and the mRNA expression of *RB1* was significantly elevated, in the Kager’s fat pad of AT patients (*n* = 30) compared to healthy CON subjects (*n* = 13). The expression of target gene mRNA was normalized to the expression of *TBP* mRNA. Normalized mRNA expression for the control subjects was set to 1. * *p* < 0.05 (compared to CON). *CS*, citrate synthase; *GLUT4*, glucose transporter 4; *ADIPOQ*, adiponectin; *RB1*, retinoblastoma 1; AT, Achilles tendinopathy; CON, control; *TBP*, TATA-binding protein.

The statistical software SAS version 9.3 was used together with SAS Enterprise Guide version 5.1 for statistical analyses and model controls. An unpaired two-tailed Student’s t-test was used to compare the control group with the AT patient group. The test was performed on the log2-transformed relative mRNA expression, which was found to be normally distributed in all cases, except for citrate synthase (*CS*), monoacylglycerol lipase (*MGL*), hormone-sensitive lipase (*HSL*), adipose triglyceride lipase (*ATGL*), and interleukin-1 receptor 1 (*IL-1R1*), for which the untransformed relative mRNA expression was normally distributed. A *p*-value of less than 0.05 was considered significant. The respective levels of significance can be found in S2 Table together with the mean relative mRNA expression values for the control group and the patient group. The mean expression value in S2 Table was back-transformed if the test was carried out on log2-transformed data. Figs 1, 2 and 3 show the untransformed mean relative mRNA expression values with SEM values. Mean relative mRNA expression for the control group was set to 1 and the other values were adjusted accordingly. The general linear model was used to test for an effect of BMI or age on gene expression levels and to test for an interaction between BMI or age and the health status (AT patient or healthy control subject).

## Results

Expression of 27 genes was analyzed in 30 AT patients and 13 control subjects. The gene targets were selected mainly to analyze three different physiological functions: inflammation, lipid and energy metabolism, and the results, stratified this way, are shown below.

### Inflammation

The mRNA expression data are shown in Fig 1 and corresponding *p*-values in S2 Table. Expression of *TNF-α*, *IL-1R1*, *IL-6*, *IL-10*, *MCP1* and the macrophage marker cluster of differentiation 68 (*CD68*) was 1.4–4.0 fold higher in AT patients when compared to the control subjects (Fig 1). The tachykinin, precursor 1 gene (*TAC1*), encoding substance P among other proteins, was detectable in 11 subjects, all of them AT patients, albeit expression in these was very low (C_t_-values 36.2–39.0). No difference between the AT and control groups was found for mRNA levels of EGF-like module-containing mucin-like hormone receptor-like 1 (*EMR1*), *IL-1β*, and resistin (*RETN*) (S2 Table).

### Lipid metabolism

The mRNA expression of *ATGL*, *HSL*, *MGL*, and acetyl-CoA carboxylase 2 (*ACC2*) was modestly but significantly 1.3–1.6 fold lower in AT patients compared to the control subjects. In contrast, carnitine-acylcarnitine translocase (*CACT*) and carnitine palmitoyltransferase (*CPT2*) showed a significantly 1.2–1.3 fold higher mRNA expression in AT patients when compared to the control subjects (Fig 2, S2 Table). No difference was found in the mRNA expression of *ACC1*, *CPT1B*, and fatty acid synthase (*FASN*), when comparing the AT patients with the control subjects (S2 Table).

### Energy metabolism, ADIPOQ and GLUT4

The mRNA expression of CS, glucose transporter 4 (GLUT4), and ADIPOQ was 1.4–3.9 fold lower, while the mRNA expression of the retinoblastoma 1 gene (RB1) was 1.3 fold higher in AT patients compared to the control subjects (Fig 3). No difference was found in the mRNA expression of leptin (LEP), glucose-6-phosphate dehydrogenase (G6PD), and uncoupling protein 1 (UCP1) when comparing the AT patients with the control subjects (S2 Table).

### The effect of BMI on gene expression

The AT patients had a higher body mass index (BMI) than the healthy controls (see Table 1). We therefore analyzed the potential effect of BMI on gene expression. Seven of the 16 genes displaying a significant differential expression between AT patients and control subjects revealed a significant effect of BMI on gene expression. These genes were *ADIPOQ*, *CACT*, *CD68*, *CS*, *GLUT4*, *IL-10*, and *IL-1R1*. To probe if the differential expression of the seven genes between the two groups was due to differences in BMI, expression levels were tested for differences between the AT patients and the control subjects when simultaneously taking BMI into account. The effect of BMI disappeared for all of the seven genes when the test included information about the health status (AT patient or control subject), and for five of the seven genes, *ADIPOQ*, *CD68*, *CS*, *GLUT4*, and *IL-10*, there was still a significant difference in expression level between AT patients and healthy controls.

### The effect of age on gene expression

On average, the AT patients were older than the healthy controls (see Table 1). Therefore, the possible effect of age on gene expression was analyzed. Expression of six of the 16 genes displaying a significant differential expression between AT patients and control subjects were significantly influenced by age. These genes were *MGL*, *RB1*, *CD68*, *CS*, *GLUT4*, and *IL-10*. To determine if the differential expression between the two groups was due to differences in age, expression levels of the six age-affected genes were tested for differences between AT patients and control subjects when simultaneously taking age into account. The possible interaction between age and health status was also tested. The effect of age disappeared for five of the six genes when the test included information about health status, and for the last gene, *RB1*, an interaction was found between age and health status. In other words, *RB1* was differentially expressed between AT patients and control subjects but the difference depended on the age of the subject. Except for *MGL*, *CD68*, and *CS*, there was still a significant difference in mRNA expression between control subjects and AT patients when accounting for age, and for the mentioned non-significant genes, the difference was still nearly significant (*p*-values 0.0561–0.0677).

## Discussion

To the best of our knowledge we here report the first investigation of gene expression changes in Kager’s fat pad of AT patients. The main finding of the present study is an increased expression of inflammatory marker genes as well as altered expression of genes involved in lipid and energy metabolism in AT patients.

### Inflammation

We have previously investigated several inflammatory markers in Achilles tendon tissue and have observed that inflammation was absent in tendon tissue of chronic AT patients [18,19]. This result concurs with the generally accepted notion that tendinopathy is an ongoing tendon degenerative process [20–22]. Nevertheless, despite the absence of tendon inflammation, corticosteroid injections are often used as a treatment, providing a short-term relief of symptoms [23]. The corticosteroid-mediated relief of symptoms is difficult to explain if tendinopathy is considered solely a degenerative process [24] and indicates that inflammation plays a role. Since it is well known that adipose tissue in itself has immunological functions [25] and has been proposed to play important roles in other tendon pathologies including Hoffa’s disease [26] and osteoarthritis [27], we considered it important to investigate the inflammatory status of Kager’s fat pad in chronic AT patients. We provide evidence for an inflammatory phenotype in the Kager’s fat pad of patients, as documented by increased expression of *TNF-α*, *IL-1R1*, *IL-6*, *IL-10*, *MCP1* and *CD68*. The existence of paracrine interactions between the inflammatory response of tenocytes in the tendon and adipocytes in the fat pad is likely. However, in the present study we analyzed the inflammatory markers of the Kager’s fat pad only, and our data therefore cannot provide definitive conclusions about this possibility. *ADIPOQ* has been suggested to be pro-inflammatory in joint diseases [16], but in our study, we find lower *ADIPOQ* mRNA levels in patients than in healthy controls, arguing against a pro-inflammatory function in AT. A previous study by Shaw *et al*. identified CD68 positive cells in the retromalleolar fat pad in rats using immunohistochemistry, and suggested that these cells might play a role combating infection and/or removal of cell debris [28]. Another reason for the presence of macrophages and inflammatory markers could be fat tissue fibrosis. It has been shown that the development of fibrosis of adipose tissue in obesity is tightly associated with tissue inflammation and the infiltration of macrophages [29] and mast cells [30]. In addition, it has been shown that increased collagen formation in the IFP might contribute to the development of synovial fibrosis [31]. Whether fibrosis is present in Kager’s fat pad of AT patients has yet to be investigated. It has been suggested that the fat pads are filling dead spaces in joints and thereby maintaining the joint cavity and promoting efficient lubrication [32]. The tip of Kager’s fat pad is associated with the Achilles tendon and minimizes pressure changes in the bursa by moving in and out of the retrocalcaneal bursa during plantar and dorsiflexion of the foot [6]. However, in case of fat pad hypertrophy, the space filling properties might contribute to symptoms of tendinopathy [33]. It has been shown previously that the size of the IFP is associated with patellar tendinopathy. Magnetic resonance imaging showed that patellar tendinopathy patients had a significantly larger fat pad compared to the control subjects when controlled for height [34]. Whether the size of Kager’s fat pad is increased in AT patients needs further investigation.

In the knee joint there is an intimate relationship between the patellar tendon and the fat pad, and a shared neurovascular supply has been observed [35]. Kager’s fat pad is related to the Achilles tendon in a similar manner [6]. In patellar tendinopathy, neurovascular infiltration into the patellar tendon has been observed [36]. Neurovascular ingrowth is associated with tendon pathology and pain. In addition, the IFP has been shown to have a nociceptive function in the anterior knee pain syndrome [37]. Shaw *et al*. also identified nerve fibers and the nociceptive marker substance P in the fat tissue [28]. Another study showed an increased number of nociceptive fibers in ‘jumpers knee’ patients [38]. Since there is growing evidence that the fat pad is involved in pain mechanisms of tendon pathologies, it is relevant to mention that expression of the *TAC1* gene, encoding substance P, was below the detection limit in most subjects of the present study. However, *TAC1* mRNA was detectable in about one third (n = 11) of the AT patients, but could not be detected in any healthy control subjects. This may indicate that Kager’s fat pad mediates pain in Achilles tendinopathy.

### Lipid and energy metabolism

In the present study, gene expression was analyzed in Kager’s fat pad only. Reduced expression of *ATGL*, *HSL* and *MGL* in Kager’s fat pad of AT patients as compared to control subjects indicates decreased lipolysis in the patient group [39]. *CACT* and *CPT2* mRNAs were increased in AT patients suggesting that the Kager’s fat pad of patients has an increased capacity for β-oxidation. By contrast, *CPT1B*, catalyzing the first and rate-limiting step of transport of fatty acids into mitochondria [40], showed no difference in expression between the AT patients and the control group. *ACC2* expression was lower in AT patients, indicative of diminished malonyl-CoA synthesis and thereby higher activity of *CPT1B* in AT patients, despite unchanged expression levels [41]. Combined, this is supportive of increased β-oxidation in Kager’s fat pad of AT patients. The gene expression analysis thus creates an apparent paradox, as lipolysis appears to be decreased, whereas fatty acid oxidation appears to increase in AT patients. Although mRNA levels need not reflect enzyme activities, one possible explanation is that in Kager’s fat pad from AT patients, more fatty acid are taken up from the circulation. This could be studied by measuring levels of fatty acid transport proteins. In AT patients, we found a decrease in expression of *GLUT4*, suggesting decreased insulin-stimulated glucose uptake in Kager’s fad pad-resident adipocytes of AT patients. Therefore, it remains possible that Kager’s fad pad of AT patients metabolizes relatively more fatty acids and less glucose than in healthy controls.

In terms of energy metabolism, we detected decreased expression of *CS* and increased expression of *RB1* in the Kager’s fat pad of AT patients compared to healthy controls. RB1 is a tumor suppressor, but is also an established inhibitor of oxidative metabolism in both white and brown adipose tissue [42,43]. Therefore, these data suggest that oxidative metabolism is lower in the fat pad of AT patients. RB1 is also inhibiting expression of the brown fat-specific *UCP1* gene [42,43]. The very low expression of *UCP1* mRNA strongly suggests that Kager’s fat pad is a white adipose tissue depot.

### The influence of BMI and age on gene expression

The possible effect of BMI and age on gene expression was analyzed. Of the 16 genes differentially expressed between the healthy control group and the AT patients, seven genes were significantly affected by BMI. For five of the seven genes, there was still a significant difference in mRNA expression between control subjects and AT patients when accounting for BMI. Thus, differences in BMI between the healthy control group and the AT patients could only explain two out of 16 differentially expressed genes. With respect to age, six of the 16 genes differentially expressed between healthy controls and AT patients were significantly influenced by age. Three of the six genes still displayed differential expression with respect to health status when accounting for age. Thus, differences in age between the healthy control group and the AT group could only explain three out of 16 differentially expressed genes.

### Limitations of the present study

A limitation of the present study is that gene expression was analyzed only by RT-qPCR. Obviously, an altered mRNA level does not necessarily reflect a change in protein level or protein activity. However, as very little tissue was available for analysis, we were limited to studying gene regulation at the mRNA level. Another limitation is that differences in cell type composition of Kager’s fat pad between healthy control subjects and AT patients might have influenced the outcome of our gene expression analysis. The inflammatory gene expression profile of AT patients most likely means an increased prevalence of immune cells in their fat pad, which again might have affected the relative expression of some of the metabolism-related genes analyzed.

## Conclusion

The contribution of Kager’s fat pad to Achilles tendinopathy is unknown. The present study reveals that Kager’s fat pad might play an important role in the pathology of disease. Expression of several inflammatory markers was up-regulated in the Kager’s fat pad of AT patients. In addition, the gene expression data are consistent with substantial alterations in lipid and energy metabolism of the fat pad of AT patients. Delineating the contribution of the inflammatory state and the altered metabolism of Kager’s fat pad to Achilles tendinopathy requires more and dedicated investigations.

## Supporting Information

S1 Table RT-qPCR primers. (DOCX)

S2 Table *p*-Values and mean expression values. (DOCX)
