# Supplementary material for: Inflammatory and Metabolic Alterations of Kager's Fat Pad in Chronic Achilles Tendinopathy
Source: PLoS One. 2015 May 21;10(5):e0127811. doi: 10.1371/journal.pone.0127811 (PMC4440827; doi:10.1371/journal.pone.0127811)
Supplement: S1 Table — (DOCX) [file pone.0127811.s001.docx]

**Table S1: RT-qPCR primers.**

| **mRNA** | **Sense primer** | **Antisense primer** |
| --- | --- | --- |
| *ACC1* | AACCAAGTAGTGAGGATGGC | GGATGTTCCCTCTGTTTGGA |
| *ACC2* | CCCCGAGAACCTCAAGAAAT | CCATGCACACGACTTTGTTT |
| *ADIPOQ* | ATGACCAGGAAACCACGACT | TGCCATCTCTGCCATCAC |
| *ATGL* | ACCAGCATCCAGTTCAACCT | ATCCCTGCTTGCACATCTCT |
| *CACT* | AAGCTGTACCAGGAGTTTGG | GGGACATCTCGCATAAGGG |
| *CD68* | CCAACAAAACCAAGGTCCAG | GGTGTCCATAGGGGAATGAG |
| *CPT1B* | AGTGACTGGTGGGAAGAGTA | GATGAGCACAAGGTCCATGA |
| *CPT2* | CCCAAACTTGAAGACACCAT | CAGGGTCCCGAAATGTAGC |
| *CS* | GGACATATCCCAACAGAGGAA | CTCTTTGCCCACTCTTTTGAG |
| *EMR1* | CTCTTCTGGGGATGTTGTGT | TATTGGTGCAGGTGGCATAA |
| *FASN* | TGCCAGAGTCGGAGAACTT | GAGGCATCAAACCTAGACAGG |
| *G6PD* | CCGAAAACACCTTCATCGTG | AGAAGGGCTCACTCTGTTTG |
| *GLUT4* | AACTGGACGAGCAACTTCATC | AGGACCGCAAATAGAAGGAAG |
| *HSL* | ATGGAAGTGCTATCGTCTCTG | AGTCAGTGGCATCTCAAAGG |
| *IL-1β* | TGATGGCTTATTACAGTGGCA | CGGAGATTCGTAGCTGGATG |
| *IL-1R1* | GACTACGTTGGGGAAGACAT | TTCCATCCTGAACAAGAGCA |
| *IL-6* | CTGGCAGAAAACAACCTGAA | CCAGGCAAGTCTCCTCATTG |
| *IL-10* | GACTTTAAGGGTTACCTGGGTT | ATGTCTGGGTCTTGGTTCTC |
| *LEP* | CTGTGCCCATCCAAAAAGTC | CTGACTGCGTGTGTGAAATG |
| *MCP1* | AAACTGAAGCTCGCACTCTC | CATTGATTGCATCTGGCTGAG |
| *MGL* | ATCGCCTATGTGACAGCAAA | GGTAGGCACCTTCATAAATCTTG |
| *RB1* | CACAAGCAACCTCAGCCTTC | GCGTTCACAAAGTGTATTTAGCC |
| *RETN* | GTGTGCCGGATTTGGTTAG | GAGGAGGAGGAGACAGAGAG |
| *TAC1* | GGCAAACGGGATGCTGATTC | GCACTCCTTTCATAAGCCATTT |
| *TBP* | CCCGAAACGCCGAATATAA | GAAAATCAGTGCCGTGGTTC |
| *TNF-α* | CCCAGGGACCTCTCTCTAAT | GCTTGAGGGTTTGCTACAAC |
| *UCP1* | CCAACTGTGCAATGAAAGTGT | CAAGTCGCAAGAAGGAAGGTA |
| *ACC1*, acetyl-CoA carboxylase 1; *ACC2*, acetyl-CoA carboxylase 2; *ADIPOQ,* adiponectin; *ATGL,* adipose triglyceride lipase; *CACT,* carnitine-acylcarnitine translocase; *CD68,* cluster of differentiation 68; *CPT1B,* carnitine palmitoyltransferase 1B; *CPT2,* carnitine palmitoyltransferase 2; *CS,* citrate synthase; *EMR1,* EGF-like module-containing mucin-like hormone receptor-like 1; *FASN,* fatty acid synthase; *G6PD,* glucose-6-phosphate dehydrogenase; *GLUT4,* glucose transporter 4; *HSL,* hormone-sensitive lipase; *IL-1β,* interleukin-1β; *IL-1R1*, interleukin-1 receptor type 1; *IL-6,* interleukin-6; *IL-10,* interleukin-10; *LEP,* leptin; *MCP1,* monocyte chemotactic protein-1; *MGL,* monoacylglycerol lipase; *RB1,* retinoblastoma 1; *RETN,* resistin; *TAC1,* tachykinin, precursor 1; *TBP,* TATA-binding protein; *TNF-α*, tumor necrosis factor-α; *UCP1*, uncoupling protein 1 | | |
