# Supplementary material for: Inflammatory and Metabolic Alterations of Kager's Fat Pad in Chronic Achilles Tendinopathy
Source: PLoS One. 2015 May 21;10(5):e0127811. doi: 10.1371/journal.pone.0127811 (PMC4440827; doi:10.1371/journal.pone.0127811)
Supplement: S2 Table — (DOCX) [file pone.0127811.s002.docx]

**Table S2: *p*-values and mean expression values.**

| **mRNA** | ***p*-value** | **Mean expression,**  **controls** | **Mean expression,**  **AT patients** |
| --- | --- | --- | --- |
| *ACC1* | 0.4985 | 0.338 | 0.384 |
| *ACC2* | 0.0249* | 10.3 | 7.18 |
| *ADIPOQ* | 0.0020* | 0.356 | 0.0909 |
| *ATGL* | 0.0327* | 105 | 81.2 |
| *CACT* | 0.0028* | 0.319 | 0.426 |
| *CD68* | 0.0001* | 0.937 | 1.96 |
| *CPT1B* | 0.0876 | 0.279 | 0.230 |
| *CPT2* | 0.0265* | 0.987 | 1.16 |
| *CS* | 0.0010* | 1.73 | 1.26 |
| *EMR1* | 0.2967 | 0.00123 | 0.000877 |
| *FASN* | 0.6863 | 22.1 | 19.9 |
| *G6PD* | 0.2790 | 0.120 | 0.136 |
| *GLUT4* | 0.0001* | 1.67 | 0.735 |
| *HSL* | 0.0077* | 27.3 | 16.8 |
| *IL-1β* | 0.0870 | 0.00728 | 0.00381 |
| *IL-1R1* | 0.0004* | 0.254 | 0.502 |
| *IL-6* | 0.0011* | 0.0118 | 0.0250 |
| *IL-10* | < 0.0001* | 0.0269 | 0.108 |
| *LEP* | 0.1112 | 9.78 | 5.49 |
| *MCP1* | 0.0024* | 0.0510 | 0.107 |
| *MGL* | 0.0271* | 2.46 | 1.58 |
| *RB1* | 0.0005* | 2.08 | 2.69 |
| *RETN* | 0.0703 | 0.0441 | 0.0652 |
| *TAC1* | - | - | 0.000265 |
| *TNF-α* | 0.0353* | 0.0179 | 0.0250 |
| *UCP1* | 0.3671 | 0.00327 | 0.00431 |
| * *p*-value < 0.05 | |  |  |
